# Fabricating retinal pigment epithelial cell sheets derived from human induced pluripotent stem cells in an automated closed culture system for regenerative medicine

**DOI:** 10.1371/journal.pone.0212369

**Published:** 2019-03-13

**Authors:** Erino Matsumoto, Naoshi Koide, Hiroko Hanzawa, Masaharu Kiyama, Mari Ohta, Junichi Kuwabara, Shizu Takeda, Masayo Takahashi

**Affiliations:** 1 Center for Exploratory Research, Research & Development Group, Hitachi, Ltd., Kobe, Hyogo, Japan; 2 Laboratory for Retinal Regeneration, RIKEN Center for Biosystems Dynamics Research, Kobe, Hyogo, Japan; 3 Planning and Development Division, Sanplatec Co., Ltd., Osaka, Japan; Newcastle University, UNITED KINGDOM

## Abstract

Regenerative medicine has received a lot of attention as a novel strategy for injuries and diseases that are difficult to cure using current techniques. Cell production, which is vital for regenerative medicine, has undergone remarkable progress via breakthroughs in developmental biology and tissue engineering; currently, cell production requires numerous experimental operators performing manual, small-scale cell cultures. Other major obstacles for cell production and regenerative medicine include the variable quality of products based on the experimental procedure, the skills of operators, the level of labor required for production, and costs. Technological developments are required to overcome this, including automation instead of manual culture. Age-related macular regeneration (AMD) is a refractory ocular disease that causes severe deterioration in central vision due to senescence in the retinal pigment epithelium (RPE). Recently, we performed an autologous transplantation of induced pluripotent stem (iPS) cell-derived RPE cell sheets and started clinical research on allografts from RPE cell suspensions differentiated from iPS cells. The use of regenerative therapies for AMD using iPS cell-derived RPE is expected to become more widespread. In the present study, human iPS cell-derived RPE cells were cultured to form RPE cell sheets using equipment with a closed culture module. The quality of the automated cultured RPE cell sheets was confirmed by comparing their morphological and biological properties with those of manually generated RPE cell sheets. As a result, machine-cultured RPE sheets displayed the same quality as manually cultured RPE sheets, showing that iPS cell-derived RPE cell sheets were successfully cultured by an automated process.

## Introduction

Regenerative medicine is an innovative type of therapy that enables the restoration of severely damaged and/or diseased tissues that would be difficult to treat with conventional methods [1]. In regenerative therapy, cell and/or tissue products are conventionally prepared using manual cell culture by skilled experimental operators, which may result in products with inconsistent quality. The production of a stable supply of uniformly high-quality products is a widespread challenge in the field of regenerative medicine.

Age-related macular degeneration (AMD) is a common disease that causes severe loss of vision in the elderly population and developed countries [2]. Atrophy or degeneration of the retinal pigment epithelium (RPE), a monolayer of pigmented cells between the neural retina and choroid layers, is thought to be a primary cause of this disease [2]. The transplantation of allogeneic RPE sheets derived from human fetuses [3,4] and autologous RPE harvesting from the peripheral region of the eye [5,6] have previously been reported as successful clinical treatments for AMD patients; however, there are major disadvantages to both forms of RPE, such as immunological rejection and invasiveness. Human pluripotent stem (hPS) cells, such as embryonic stem cells and induced pluripotent stem (iPS) cells, are a promising source for the development of cell-based regenerative therapies because they can be used to produce a broad spectrum of human cell types *in vitro* without limit. Therefore, RPE derived from hPS cells has emerged as an ideal alternative tissue source [7]. Previously, we developed a method for the generation of iPS cell-derived RPE cell sheets [8] and reported the successful autologous transplantation of an RPE cell sheet differentiated from iPS cells generated from skin fibroblasts in a patient with AMD; the results showed that the transplanted sheet remained intact and functioned for one year after surgery [9].

At present, hPS cells and functional cells derived from hPS cells are mostly generated and cultured manually at a small scale and with variable quality for individual patients in clinical research and treatment [10]. Automated cell culture systems are expected to replace manual culture methods, and the development of such systems has been motivated by clinical and industrial demand [11–15]. Currently, “open” automated cell culture systems are the most common [13–15]. They are designed to follow an expert’s manual operation of processes in a laboratory, including cell seeding, replacing medium, and harvesting, using robotics and automation technology. It was reported that iPS cells were successfully proliferated through automation for long-term cultivation with multiple passages using an open system while maintaining pluripotency and the capability to differentiate into specific cell types [15]. However, there are still several risks associated with cultivation using open systems, such as contamination and uncontrolled changes during proliferation; each time the culture medium is replaced or cultured cells are harvested, the system must be opened and exposed to the environment. Therefore, we have attempted to develop a “closed” automated culture system in which cells are never directly exposed to the external environment during the procedure; using this system, we previously achieved the automated generation and culture of corneal epithelial cell sheets [16,17] and oral mucosal epithelial cell sheets (Nishimura A, Submitted) in accordance with established manual laboratory culture protocols (S1A Fig). Our closed system is comprised of several distinct features. All components, including the culture vessels, bags to supply medium, disposal units, and tubes, are connected before performing the protocol (S1B Fig). All connected components in the culture module are sealed or attached with sterile connections. The module is then pre-sterilized for aseptic processing. All necessary substances for targeted cell culture, including CO_2_, O_2_, and media with nutrients, are supplied through automated and accurate flow control mechanisms using peristaltic pumps and pinch valves. To avoid cross-contamination between cultivation batches, the culture modules are disposable and single-use. From the standpoint of good manufacturing practice, a closed system is considered to be more suitable than an open system because of increased sterility and safety [16–19].

In the present study, human iPS cell-derived RPE was grown using our latest cell culture equipment with a closed culture system, the ACE3 system, to determine the feasibility of the machine culture of RPE cell sheets.

## Materials and methods

### Cell strain and culture of RPE cell sheets

Human RPE (hRPE) (#00194987) was purchased from Lonza (Basel, Switzerland). A previously established human iPS (hiPS: cell line 253G1) cell-derived RPE line [8] was provided by the RIKEN Center for Biosystems Dynamics Research (Hyogo, Japan; formerly known as the Center for Developmental Biology). In this paper, RIKEN will be referred to as the original site, while the Hitachi laboratory (Hyogo, Japan) will be referred to as the satellite site; most experiments were conducted at the satellite site, including machine culture with the ACE3 system and control experiments with manual culture. For research purposes, all culturing procedures for the manual and machine culture of RPE cell sheets described in this study were conducted in a conventional cell culture laboratory, and not in a cell processing center. hRPE and hiPS-RPE cell sheets were generated as previously described by Kamao et al., with minor modifications [8]. A collagen gel solution was prepared using beMatrix Low Endotoxin Collagen AT Solution (Nitta Gelatin, Osaka, Japan). The solution, along with 5× DME and reconstitution buffer (Nitta Gelatin), were mixed at a ratio of 7:2:1 on ice with gentle shaking, added to a 12-well Transwell polyester membrane cell culture insert (#3460, Corning Inc., Corning, NY, USA), and incubated at 37 °C for 30 min. hRPE and hiPS-RPE (5 × 10^5^ cells/0.6 mL) were seeded on the gel and cultured in RPE sheet medium containing Nutrient Mixture F-10 Ham (#N6908, Sigma Aldrich, St. Louis, MO, USA) and 10% fetal bovine serum (#12007, SAFC Bioscience, Lenexa, KS, USA); the RPE sheet medium was added to the basal side of the insert (1.5 mL/well). After 14 days, the hRPE and hiPS-RPE culture media were replaced with a serum-free RPE medium containing DMEM (#D6046), 30% F12 Ham (#N6658, Sigma Aldrich), 2% B27 (#F17504, Gibco, Thermo Fisher Scientific, Waltham, MA, USA), 1% L-glutamine (G#7513, Sigma Aldrich), 1% penicillin-streptomycin (#15140, Gibco), 10 ng/mL basic fibroblast growth factor (bFGF; #064–05381, Wako Pure Chemical Industries, Osaka, Japan), and 0.5 μM SB431542 (#S4317, Sigma Aldrich). The media on the apical side (0.6 mL) and on the basal side (1 mL) were changed every 3–4 days throughout the protocols.

### Automated cell culture system

hRPE and hiPS-RPE sheets were machine-cultured using the ACE3 automated cell culture system (prototype, Hitachi). A maximum of ten RPE sheets can be grown simultaneously using ACE3. A culture module composed of culture medium bottles, closed cell culture vessels, and bags for waste fluids were connected with tubes. The ends of the tubes were capped using disc filters (pore size: 0.22 μm, PureFlo 25 mm Disc Filter, Saint-Gobain, Courbevoie, France) or sterile connectors (AseptiQuick G, Colder Products Company, St. Paul, MN, USA). The assembly of modules was conducted in a sterile booth to avoid contamination. Each module was sterilized by 15-kGy gamma-ray irradiation. Before culturing, a sterilized culture module was installed and a pressure test was carried out using the ACE3 to ensure there were no leaks. Cells suspended in hiPS-RPE or hRPE culture medium were prepared from a frozen cell stock and manually transferred into the cell suspension bottles. Thereafter, the cell suspension bottles and culture medium bottles were connected to the module through sterile connectors. Because the standard closed culture vessels used for the ACE3 are 6-well cell-culture inserts, a silicon-based adapter for 12-well cell-culture inserts (S1C Fig, Sanplatec Co., Ltd., Osaka, Japan) was designed and attached to the culture vessels. Next, a portion of the cell suspension was automatically loaded onto the inserts, followed by automated culture for 48 days. The closed cell culture vessel was supplied with humidified air containing 5% CO_2_ as well as culture medium. The air was passed through a 0.22-μm filter and supplied into the vessels at a flow rate of 70 mL/min per one vessel every 30 min for 2 min intervals. The inside of the ACE3 incubator was kept in the dark at 37 °C. A fixed quantity of culture medium was pumped from a medium bottle installed in the refrigerator of the ACE3, then preheated and incubated for the indicated time periods at 37 °C. Next, 0.6 mL culture medium was added to the apical side of the insert and 1.1 mL culture medium was added to the basal side after removing an equal volume of media from both sides at a flow rate of 9 mL/min. This process was repeated every 3–4 days.

After 48 days of hRPE and hiPS-RPE cell sheet culturing, the closed cell culture vessels were detached from ACE3 by heat welding and cutting the tubing aseptically. Media were then removed from both sides of each well and replaced with 0.6 mL and 1.5 mL fresh culture medium in the apical and basal sides of the insert, respectively. After replacing the media, the cell sheets were cultured for another 24 h in a conventional CO_2_ incubator. Then, the same volumes of culture media were collected as samples for ELISA. The media was then replaced at the same volumes described above for transepithelial electrical resistance (TER) measurements. Finally, hRPE and hiPS-RPE cell sheets were recovered from the collagen gels by collagenase I (#05172969103, Roche, Basel, Switzerland) treatment and washed with PBS for further analysis using immunohistochemistry and real-time PCR. In each analysis listed above, at least three replicates were used.

### Immunohistochemistry

After hRPE and hiPS-RPE cell sheets were cultured for 49 days, the insert membrane was removed and the generated hRPE and hiPS-RPE cell sheets were peeled off and fixed in Superfix (#KY-500, Kurabo, Osaka, Japan) at 4 °C for 10 min. The RPE sheets were washed in PBS and cryoprotected in PBS supplemented with 10% sucrose at 4 °C for 1 h followed by incubation overnight at 4 °C in PBS supplemented with 30% sucrose. The RPE cell sheets were rapidly frozen in Tissue-Tek OCT compound (#4583, Sakura Finetek Japan, Tokyo, Japan), and the frozen cell sheet samples were sectioned at a thickness of 10 μm. Specimens were permeabilized with 0.2% Triton X-100 (#93443, Sigma) in PBS at room temperature for 30 min, washed with PBS, and blocked using Blocking One (#03953, Nacalai Tesque, Kyoto, Japan) reagent at room temperature for 1 h; the specimens were then incubated with primary antibodies (ZO-1 polyclonal antibody: #61–7300, 1:200 dilution; ZO-1 monoclonal antibody: #33–9100, 1:1000, Thermo Fisher Scientific; anti-collagen IV antibody: #ab6311, 1:400; anti-laminin antibody: #ab11575, 1:200; Na, K-ATPase antibody: #ab7671, 1:100; MERTK antibody: #ab52968, 1:100; RPE65 antibody: #ab13826, 1:250; PMEL17 antibody: #ab137078, 1:500, Abcam, Cambridge, UK; Claudin19 antibody: #sc-365967, 1:250, Santa Cruz Biotechnology, Inc. Texas, USA) at 4 °C overnight. Signals were detected using secondary antibodies labeled with Alexa Fluor 488 and 546 after incubation for 1 h at room temperature.

### ELISA for vascular endothelial growth factor (VEGF) and pigment epithelium derived factor (PEDF)

The concentrations of PEDF and VEGF in the collected samples were first determined using kits for PEDF Human ELISA (#RD191114200R, BioVendor, Brno, Czech Republic) and Human VEGF-A Platinum ELISA (#BMS277, eBioscience, Waltham, MA, USA), respectively, according to the manufacturers’ instructions. Then, the obtained values were multiplied by the volume of the medium to calculate the total absolute amount of PEDF and VEGF secreted by each RPE cell sheet into the medium in wells on the apical or basal side of the insert. The polarity of PEDF or VEGF was described as the ratio of the absolute quantity secreted into the medium on the apical side of the insert to that on the basal side of the insert.

### Real-time PCR analysis

The collected hRPE and hiPS-RPE cell sheets were homogenized using QIAshredder (#79654, Qiagen, Hilden, Germany), and total RNA was extracted using the RNeasy Plus Micro kit (#74034, Qiagen) according to the manufacturer’s instructions. The RNA concentration was measured using a NanoDrop 2000 spectrophotometer (Thermo Fisher Scientific). A reverse transcription reaction solution was prepared using the SuperScript III CellsDirect cDNA Synthesis Kit (#18080200, Invitrogen, Carlsbad, CA, USA), and cDNA was synthesized at 25 °C for 15 min, 50 °C for 60 min, and 70 °C for 15 min. The samples were then stored at 4 °C. Real-time PCR was performed using six RPE-specific gene primers (*RPE65*: Hs01071462_m1, *CRALBP*: Hs00165632_m1, *MERTK*: Hs01031979_m1, *BEST1*: Hs04397293_m1, *Claudin19*: Hs00961709_m1, *Claudin11*: Hs00194440_m1), and *GAPDH* (Hs02786624_g1, Thermo Fisher Scientific) with 40 cycles of 95 °C for 5 s and 60 °C for 30 s.

### Transepithelial electrical resistance (TER)

TER was measured using an electrical resistance system (Millicell ERS-2, Merck Millipore, Darmstadt, Germany). The test electrode was connected to the input port on the system, and the meter was adjusted to 1000 Ω. The electrode was washed with 70% ethanol for 15 min and rinsed with serum-free RPE medium for 15 min. The TER values of the hRPE and hiPS-RPE cell sheets on the insert were then measured by the electrode. These were determined by subtracting the background value of the insert without cells on the collagen gel from the experimental values. Then, the TER value was calculated by multiplying by the unit of area (cm^2^), which was corrected by the surface area of the insert.

### Statistical processing

A statistical analysis of the acquired experimental data was carried out using JMP 13 (SAS Institute Inc., Cary, NC, USA). Statistical significance (P <0.05) was examined by Steel’s test for multiple comparisons.

## Results

The primary goal of this study was to achieve the machine-based culture of iPS cell-derived RPE cell sheets in a closed, automated cell culture system. Initially, however, we used hRPE cells with a manual cell culture protocol that could be easily converted to a machine-operated protocol. Once this process was established, we then set out to confirm whether the machine culture of hiPS RPE cell sheets was viable through a proof-of-concept experiment. For comparison, hRPE was seeded from the same batch of cell suspensions to perform both culturing methods simultaneously. After 49 days of culture, the generated hRPE cell sheets were detached from the inserts, and a series of frozen vertical thin sections was prepared. Immunohistochemistry was performed on these sections using specific antibodies against ZO-1, a tight junction-related factor; as well as laminin and type IV collagen, extracellular matrix proteins found in the basement membrane (S2 Fig). Phase-contrast images of the hRPE cell sheets generated by the ACE3 system showed a partially multilayered, rather than monolayered, conformation (S2A and S2B Fig). Fluorescence signals from laminin and type IV collagen were observed between RPE layers. Fluorescence signals from ZO-1 were observed outside and/or between cells with similar morphological images (S2C–S2F Fig), as previously reported [20]. Phase-contrast and fluorescence images obtained from the manually cultured hRPE cell sheets showed similar results to those obtained from the machine-cultured hRPE cell sheets (S2G and S2H Fig). Fluorescence from laminin and type IV collagen was also identified between RPE layers (S2I–S2L Fig).

Quantitative analysis of the secreted proteins PEDF and VEGF was performed by ELISA on samples collected from the apical and basal media of cultured hRPE (S1 Table). The total amount of PEDF in the culture medium collected from the apical side of the insert for machine-cultured hRPE was greater than that in the culture medium collected from the basal side. In contrast, the total amount of VEGF in the culture medium collected from the basal side of the insert was greater than that in the culture medium collected from the apical side. This suggests that hRPE cultured by the ACE3 exhibited polarity, as previously reported [8,21,22]. In addition, the differences in the amounts of PEDF and VEGF were similar for the collected media obtained from the manually cultured hRPE.

RPE-related gene expression (*BEST1*, *RPE65*, *MERTK*, *CRALBP*, *Claudin19*, and *Claudin11*) [8,21,23,24] was evaluated by real-time PCR in samples extracted from the hRPE cell sheets cultured by the ACE3 (S3 Fig). These markers (*BEST1*, *RPE65*, *MERTK*, *CRALBP*, *Claudin19*, and *Claudin11*) were confirmed to be expressed, and their expression levels were not significantly different between samples obtained from manually cultured and machine-cultured hRPE cell sheets.

The TER value, which is used to determine cell density and analyze the formation of the intercellular adhesion structure of cell sheets, was determined in the hRPE cell sheets generated by the ACE3 after 49 days of culturing (S4 Fig). The mean TER value of the cell sheets generated by the ACE3 was 161 ± 90 Ω·cm^2^, while that of the cell sheets generated by manual culture was 147 ± 133 Ω·cm^2^. No statistically significant differences were found between the machine- and manually cultured sheets.

**Fig 1:**
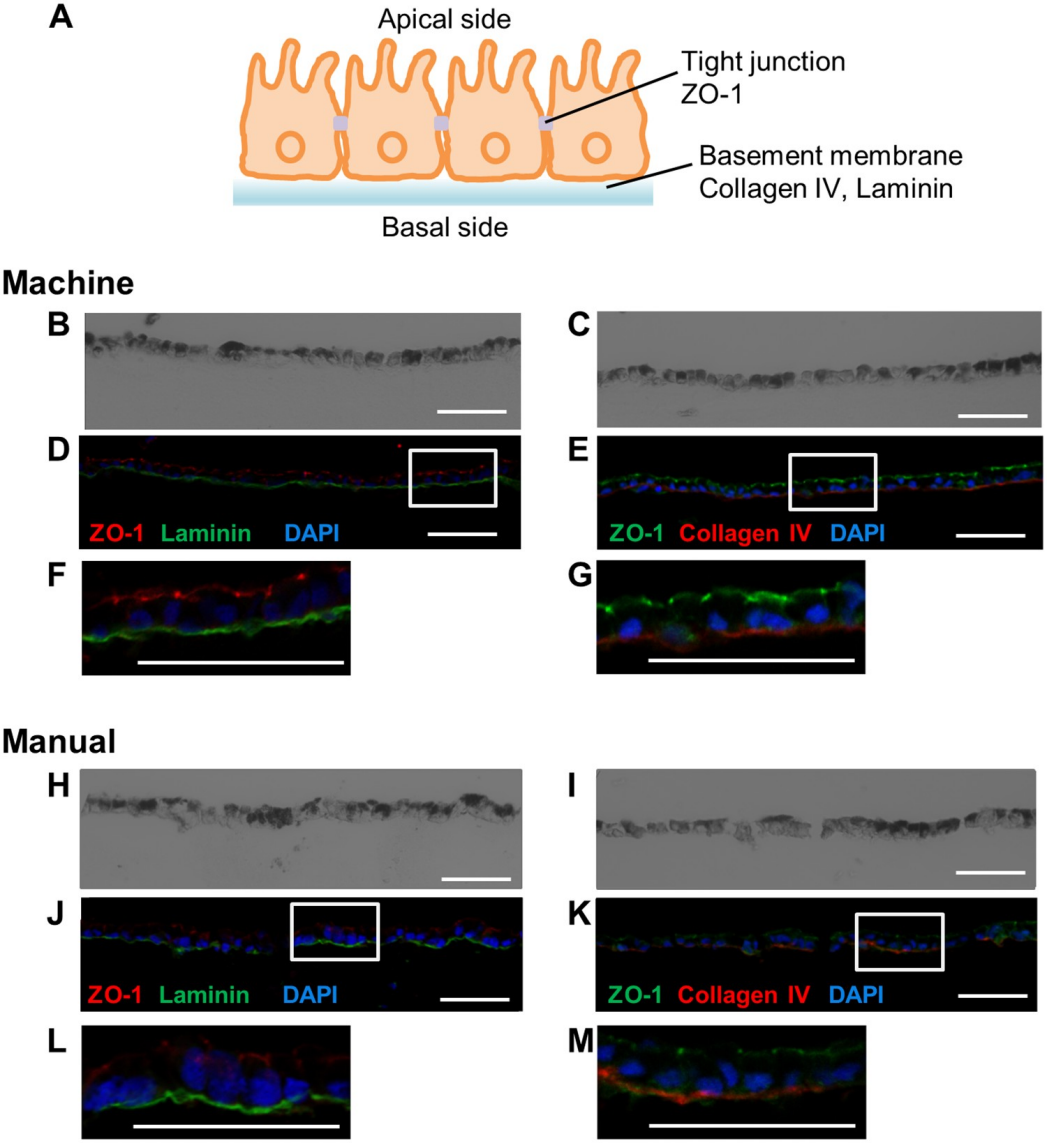
Phase-contrast and fluorescence images of immunostaining. hiPS-RPE cell sheets were cultured for 49 days using both machine and manual culture methods. (A) Schematic figure showing a cross-section of the RPE cell sheet. (B–G) Phase-contrast (B, C) and fluorescence (D–G) images of machine-cultured hiPS-RPE cell sheets. (B, D, F) Immunostaining for tight junction (ZO-1, red) and basement membrane (laminin, green) proteins. (C, E, G) Immunostaining for tight junction (ZO-1, green) and basement membrane (type IV collagen, red) proteins. (H–M) Phase-contrast (H, I) and fluorescence (J–M) images of manually cultured hiPS-RPE cell sheets. (H, J, L) Immunostaining for tight junction (ZO-1, red) and basement membrane (laminin, green) proteins. (I, K, M) Immunostaining for tight junction (ZO-1, green) and basement membrane (type IV collagen, red) proteins. Scale bars: 50 μm.

Next, we performed a proof-of-concept study using hiPS-RPE cells. For comparison, hiPS-RPE was seeded and cultured using both the machine and manual culture methods from the same batch of hiPS-RPE cells. After 49 days of culturing, the machine-cultured hiPS-RPE cell sheets were removed from the insert. The resultant hiPS-RPE cell sheets were more fragile than those derived from hRPE, and several sheets broke apart when they were detached. Serially frozen vertical thin sections were prepared for immunohistochemistry (Fig 1) using specific antibodies against ZO-1, laminin, and type IV collagen. Fluorescence signals derived from ZO-1, laminin, and type IV collagen were observed in the hiPS-RPE cell sheets cultured by the ACE3 (Fig 1B–1G). Fluorescence signals were also observed in the manually cultured hiPS-RPE cell sheets (Fig 1H–1M). However, several missing cells were observed in the images obtained from the manually cultured hiPS-RPE cell sheets (Fig 1H–1K).

**Fig 2:**
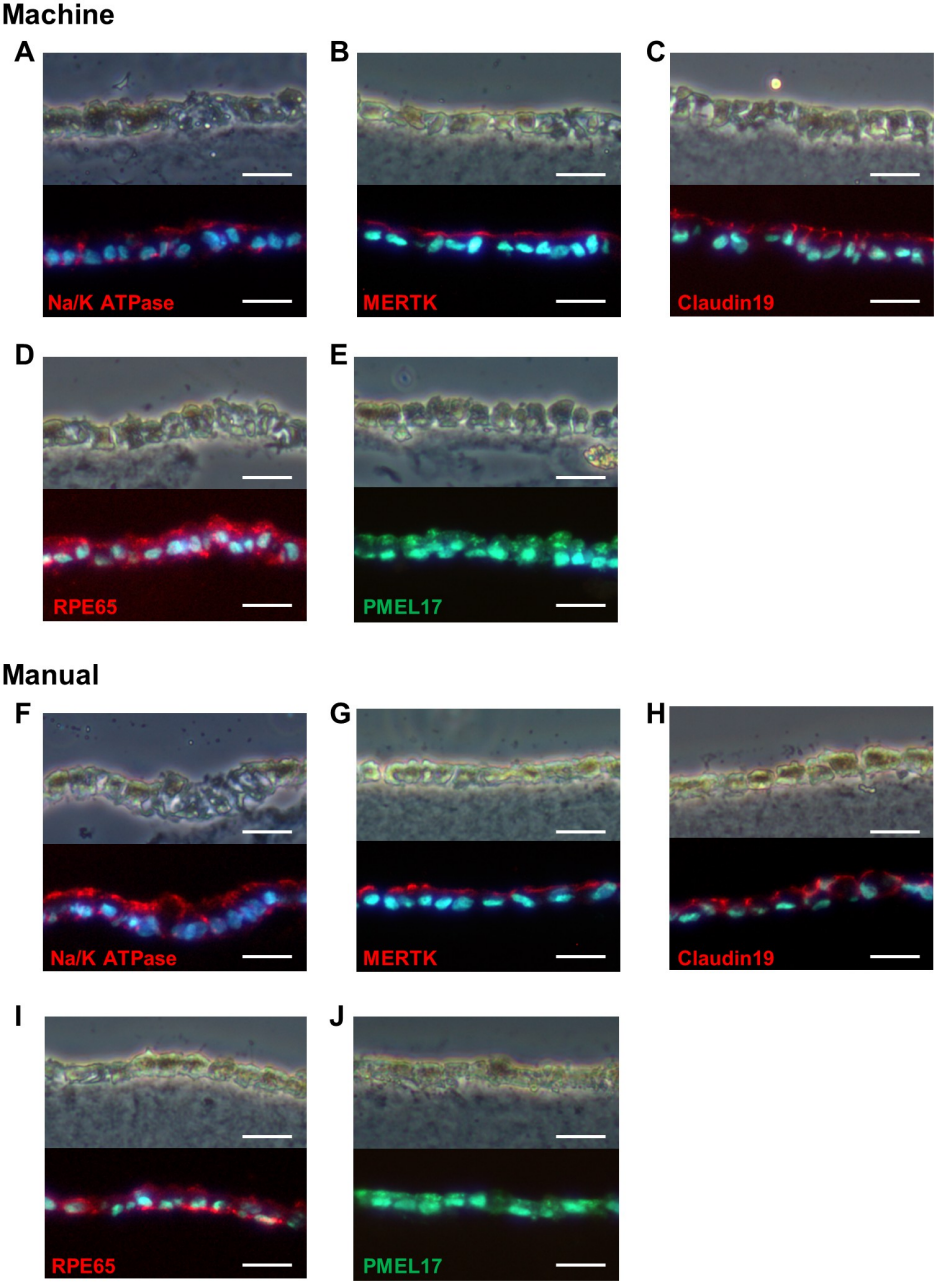
Phase-contrast and fluorescence images of immunostaining. hiPS-RPE cell sheets were cultured for 49 days using both machine and manual culture methods. (A–E) Phase-contrast image (top of each figure) and corresponding fluorescence image (bottom of each figure) of vertical sections of machine-cultured hiPS-RPE cell sheets. (A) Immunofluorescence detection of Na/K ATPase, (B) MERTK, (C) Claudin19, (D) RPE65, and (E) PMEL17. (F–J) Phase-contrast image (top) and corresponding fluorescence image (bottom) of vertical sections of manually cultured hiPS-RPE cell sheets. (F) Immunofluorescence detection of Na, K ATPase, (G) MERTK, (H) Claudin19, (I) RPE65, and (J) PMEL17. Nuclei were stained with DAPI. Scale bars: 20 μm.

In addition, as shown in Fig 2, immunohistochemical detection of the other RPE marker proteins, Na/K ATPase, MERTK, Claudin19, RPE65, and PMEL17 was performed using specific antibodies. As a result, the signals obtained from antibodies specific for RPE marker proteins were not significantly different between the hiPS-RPE cell sheets fabricated by machine and manual culture, and they showed the similar localization, as previously reported [23,25,26].

The total amounts of secreted PEDF and VEGF in the culture media of hiPS-RPE collected from the apical and basal sides of the insert were determined by ELISA (Table 1). The amount of PEDF in the culture medium collected from the apical side of the insert for both the manually and machine-cultured hiPS-RPE was greater than that in the culture medium collected from the basal side. Conversely, the amount of VEGF in the culture medium collected from the basal side of the insert was greater than that in the culture medium collected from the apical side. These results suggest that hiPS-RPE cultured by both machine and manual methods exhibits polarity, as previously reported [8,21,22].

**Table 1:** Amount of proteins secreted into media of hiPS-RPE cell sheets over 24 h at 48 days after seeding.

	Amount of PEDF and VEGF (mean ± SD)		
Culture Method		PEDF (μg)	VEGF (ng)
Machine (n = 9)	Apical	1.12 ± 0.21	6.84 ± 2.88
	Basal	0.58 ± 0.45	11.39 ± 2.28
	Apical/Basal	2.66 ± 1.33	0.57 ± 0.16
Manual (n = 7)	Apical	0.67 ± 0.13	10.63 ± 2.45
	Basal	0.27 ± 0.04	15.78 ± 3.24
	Apical/Basal	2.48 ± 0.46	0.72 ± 0.25

PEDF: pigment epithelium derived factor, VEGF: vascular endothelial growth factor.

**Fig 3:**
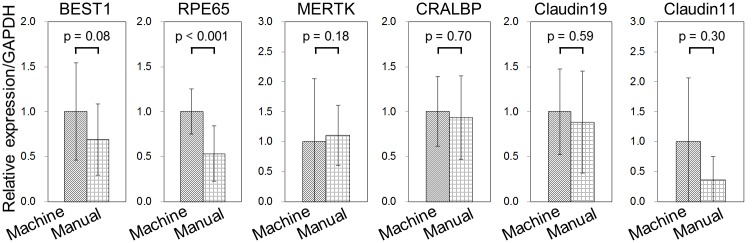
Real-time PCR analysis of RPE-related gene expression in hiPS-RPE cell sheets. Data were obtained from two independent experiments. Machine cell culture, n = 7, manual cell culture, n = 5. All data are represented as the means ± SD.

RPE-related gene expression (*BEST1*, *RPE65*, *MERTK*, *CRALBP*, *Claudin19*, and *Claudin11*) in samples extracted from the manually and machine-cultured hiPS-RPE cell sheets was determined by real-time PCR (Fig 3) [8,21,23,24]. The markers were all expressed in both samples. There was no significant difference in the expression of any marker, except RPE65, which showed significantly higher expression in the machine-cultured samples.

**Fig 4:**
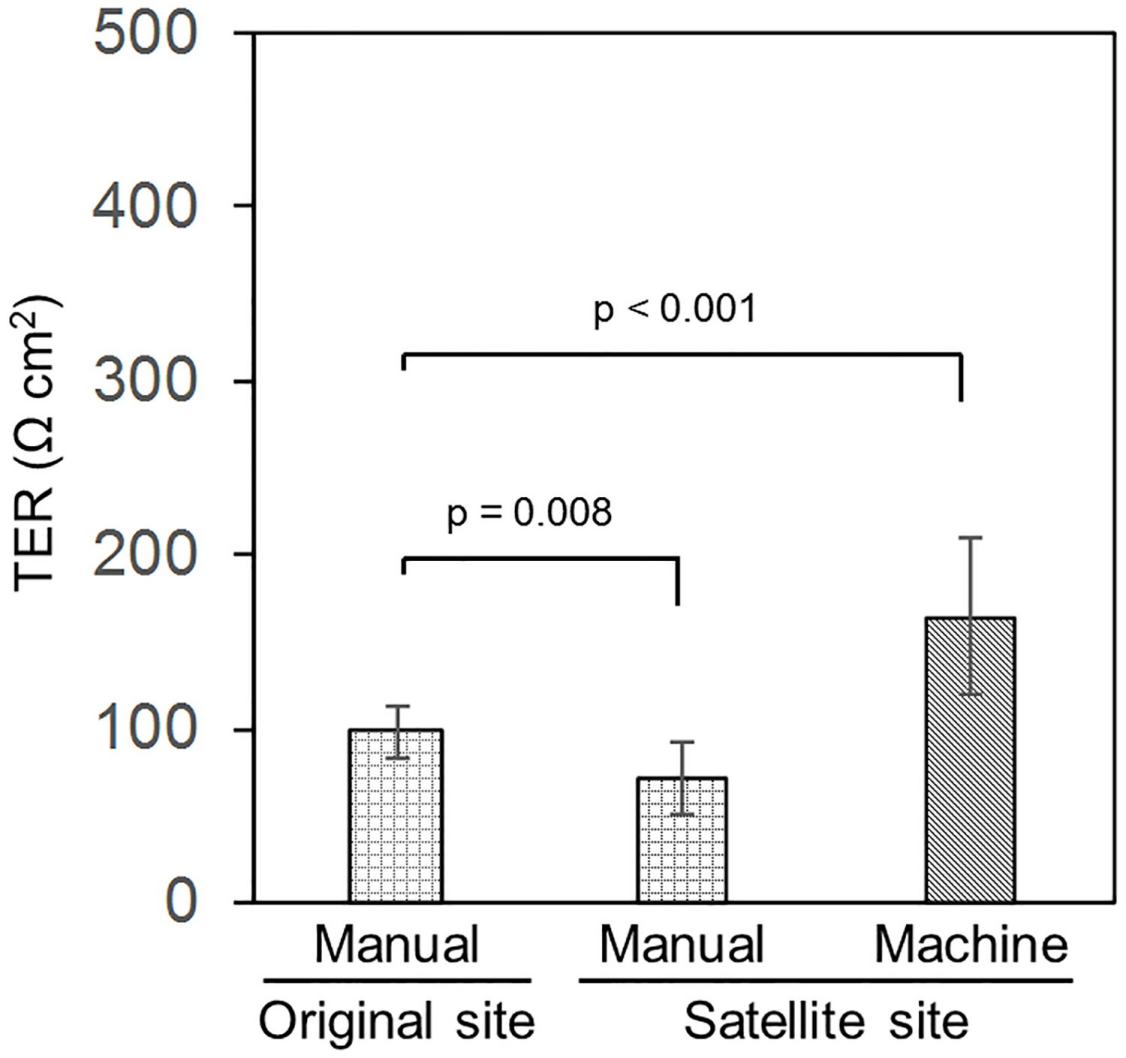
TER values of hiPS-RPE cell sheets cultured by machine and manually. hiPS-RPE cell sheets were cultured manually or using the automated ACE3 system and analyzed 49 days after seeding. The TER values of the hiPS-RPE cell sheets were calculated by subtracting the value of inserts covered with collagen gels as a blank from those of the experimental inserts. Data were obtained from two independent experiments. Machine cell culture, n = 9, manual cell culture, n = 7. All data are represented as the means ± SD.

The TER values of the manually and machine-cultured hiPS-RPE cell sheets were measured after 49 days of culturing, and each value was compared with the previously determined TER value of the hiPS-RPE cell sheets at the original site (Fig 4). The mean TER value of the cell sheets cultured by the ACE3 was found to be 164 ± 45 Ω·cm^2^, while that of the cell sheets cultured manually was 71 ± 21 Ω·cm^2^. Furthermore, the mean TER value of the hiPS-RPE cell sheets cultured manually at the original site was 98 ± 15 Ω·cm^2^. Statistical analysis using a nonparametric Steel test showed that the TER value of the machine-cultured hiPS-RPE cell sheets was significantly higher than that of the manually cultured cell sheets.

## Discussion

In this study, to verify the feasibility of fabricating RPE cell sheets using machines, hRPE and hiPS-RPE were cultured in a closed system. In this system, a sterile culture module was used; the module was a single-use unit composed of tubing, a culture medium bottle, closed cell-culture vessels, and bags for waste fluid. We found that RPE cell sheets cultured by the ACE3 showed similar characteristics to those of cell sheets cultured manually, suggesting that the machine fabrication was successful.

Through immunohistochemical analysis of the machine-cultured RPE cell sheets, it was found that tight junctions and basement membranes, which are important characteristics of RPE cell sheets, formed as efficiently as in the manually cultured cell sheets. In addition, it was found that Na/K-ATPase and MERTK proteins were expressed at the basolateral side of the cell sheets, Claudin19 was localized to tight junctions, and the proteins ZO-1, RPE65, and PMEL17 were properly expressed, which supports the belief that the hiPS-RPE cell sheets cultured by machine were morphologically and functionally similar to those cultured manually. However, multiple layers of RPE were sometimes observed in both the machine-cultured and manually cultured hRPE cell sheets (S2 Fig), which was inconsistent with a previous study on hiPS-RPE cell sheets cultured manually *in vitro* [8]. In future studies, the reproducibility of these results must be verified by increasing the number of cell-sheet samples and by performing core experiments such as time-course studies to analyze whether there is a relationship between RPE cell growth and cell sheet formation.

Analysis of the secretion of PEDF and VEGF by ELISA showed the same tendencies reported previously [8]; PEDF was primarily secreted into the culture medium, while VEGF showed lower levels of secretion on the apical side of the insert than those on the basal side in both manually and machine-cultured RPE cell sheets. These patterns of RPE-related protein secretion into the culture medium during RPE culture are known as RPE polarity, and the above results suggest that cell sheets cultured using both methods formed the characteristic structures of RPE, as expected [8].

Next, we analyzed the gene expression of six RPE-related genes. We found that they were expressed in hRPE and hiPS-RPE cell sheets cultured using both methods, confirming that the sheets maintained their previously reported characteristics [8]. We also found that their expression levels were not significantly different between cell sheets cultured using either method, with the minor exception of RPE65 in the hiPS-RPE cell sheets. The reproducibility of these results will be verified by increasing the number of samples in future studies.

It is important to determine the TER value, as it is a common index of the confluence and density of RPE cell sheets [27]. The TER value is thought to represent ion flow in the epithelial layer. A high TER value is considered to correlate well with high cell density and high barrier function in which characteristic features, such as a tight junction structures and polarization of RPE cell sheets, are maintained [27]. A typical TER value for RPE obtained *in vivo* was reported to be approximately 150 Ω·cm^2^, and TER values for an RPE cell sheet generated *in vitro* ranged from approximately 25–500 Ω·cm^2^ [27]. Kamao et al. reported the first manually cultured hiPS-RPE cell sheet, for which the TER value was 167 ± 23 Ω·cm^2^ after 49 days of culturing [8]. In the present study, the TER values of the hRPE and hiPS-RPE cell sheets after 49 days of machine culturing were 161 ± 90 and 164 ± 45 Ω·cm^2^, respectively; these values were similar to those previously reported [8]. This suggests that RPE cell sheets were successfully cultured using a machine in a closed system in terms of their quality. The TER value of the manually cultured hiPS-RPE cell sheets was 71 ± 21 Ω·cm^2^, significantly lower than that of the machine-cultured cell sheets. The TER value of the hiPS-RPE cell sheets from the same lot manually cultured at the original site was 98 ± 15 Ω·cm^2^. The reason for this low TER value may have been due to the specific cell lot, but this is unclear.

As the above results indicate, machine culture allows for the consistent culture of RPE cell sheets without depending on operational skills and/or culturing facilities. Measuring the TER value is a simple and convenient method for the nondestructive evaluation of generated RPE cell sheets; however, it is difficult to evaluate partial and localized differences in a generated cell sheet structure using this method. Furthermore, immunohistochemical analyses make it possible to precisely verify structures and features in small and restricted areas of generated RPE cell sheets. For the clinical application of RPE cell sheets, it will be necessary to choose suitable areas on a generated cell sheet for transplantation and to process those into small pieces using the appropriate evaluation processes. Combining RPE culture using automated equipment and a novel platform for evaluating generated RPE cell sheets with a noninvasive and label-free method will strongly support the further spread of regenerative therapies.

**Fig 5:**
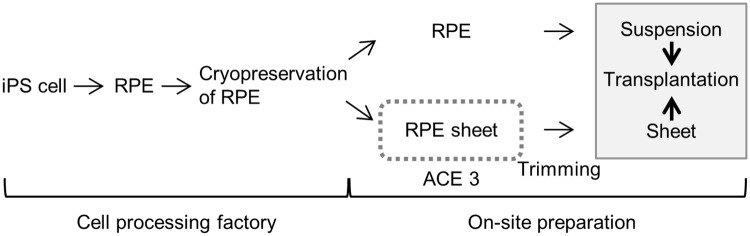
Perspective and potential applications of the present study.

Regenerative medicine has brought therapeutic innovations that have allowed damaged tissues or organs to be replaced where it was previously not possible. As research progresses, regenerative therapy using iPS cell-derived RPE for AMD is expected to become more widespread. Currently, the procedure for fabricating RPE cell sheets comprises two main steps, both of which are time- and labor-intensive when performed with manual culture. First, iPS cells are amplified and differentiated into RPE cells that are frozen as an RPE cell stock. Second, RPE cell sheets are generated and trimmed for transplantation (Fig 5). The first step requires the large-scale production and preparation of frozen cell stocks. The second step requires on-site individualized processing. Cell sheets are produced at an individual scale for each patient at the hospital where the transplantation is to be conducted. When transplanting RPE cell sheets in patients with AMD, RPE cell sheets of sufficient quality and quantity should be transplanted as soon as possible to improve the efficacy of the treatment. Additionally, it is difficult to produce cell sheets with consistently high quality through on-site fabrication, because this requires intensive maintenance of the facilities/equipment, as well as well-trained and skilled experimental operators to perform the culture protocols; the strict regulation of cell production would therefore become a burden for the hospital. For this reason, the continuous development of automation technology for the fabrication of RPE cell sheets is required to overcome these issues.

## Conclusions

In this study, we confirmed that it is possible to fabricate RPE cell sheets, especially hiPS-RPE cell sheets, using a closed automated cell-culture system, the ACE3, thus removing the need for operational skills and facilities. In the future, this automated cell-culture equipment will potentially play an important role in cell production for regenerative therapy and will enable the stable culture of high-quality RPE cell sheets in all hospitals.

## Supporting information

S1 Fig The automated cell culture equipment, ACE3 (Prototype, Hitachi), used in this study. (A) Layout of ACE3. (B) Schematic diagram of medium/gas flow in the closed cell culture system. (C) Configuration of the closed cell culture vessel. Left: Silicon-based adapter for insert produced for present study. Right: Parts structure.
(TIF)

S2 Fig Phase-contrast and fluorescence images of immunostaining. (A–F) Phase-contrast (A, B) and fluorescence (C–F) images of machine-cultured hRPE cell sheets cultured with ACE3. (A, C, E) Immunostaining for tight junction (ZO-1, red) and basement membrane (laminin, green) proteins. (B, D, F) Immunostaining for tight junction (ZO-1, green) and basement membrane (type IV collagen, red) proteins. (G–L) Phase-contrast (G, H) and fluorescence (I–L) images of manually cultured hRPE cell sheets. (G, I, K) Immunostaining for tight junction (ZO-1, red) and basement membrane (laminin, green) proteins. (H, J, L) Immunostaining for tight junction (ZO-1, green) and basement membrane (type IV collagen, red) proteins. Scale bars: 50 μm.
(TIF)

S3 Fig Real-time PCR analysis of RPE-related genes in hRPE cell sheets. Machine cell culture, n = 5, manual cell culture, n = 4. All data are represented as the means ± SD.
(TIF)

S4 Fig TER value of machine- and manually cultured hRPE cell sheets 49 days after seeding. The TER values of the hRPE cell sheets were calculated by subtracting the value from inserts covered with collagen gels as a blank from those of the experimental inserts. Machine cell culture, n = 12, manual cell culture, n = 11. All data are represented as the means ± SD.
(TIF)

S1 Table Amount of proteins secreted into media of hRPE cell sheet over 24 h at 48 days after seeding. (TIF)
