# Supplementary figures and images for: Fabricating retinal pigment epithelial cell sheets derived from human induced pluripotent stem cells in an automated closed culture system for regenerative medicine
Source: PLoS One. 2019 Mar 13;14(3):e0212369. doi: 10.1371/journal.pone.0212369 (PMC6415881; doi:10.1371/journal.pone.0212369)

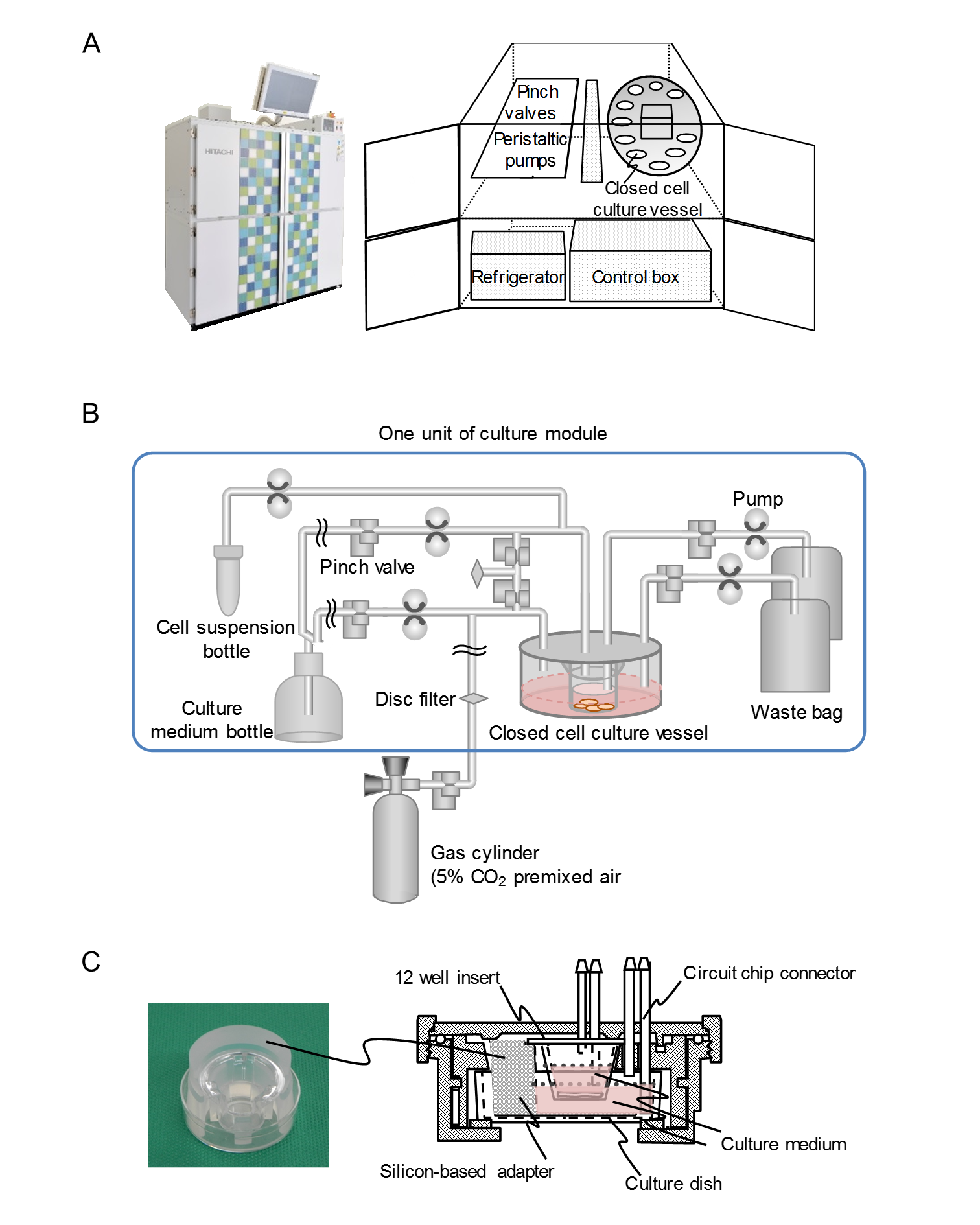

Supplement: S1 Fig — (A) Layout of ACE3. (B) Schematic diagram of medium/gas flow in the closed cell culture system. (C) Configuration of the closed cell culture vessel. Left: Silicon-based adapter for insert produced for present study. Right: Parts structure. (TIF) [file pone.0212369.s001.tif]

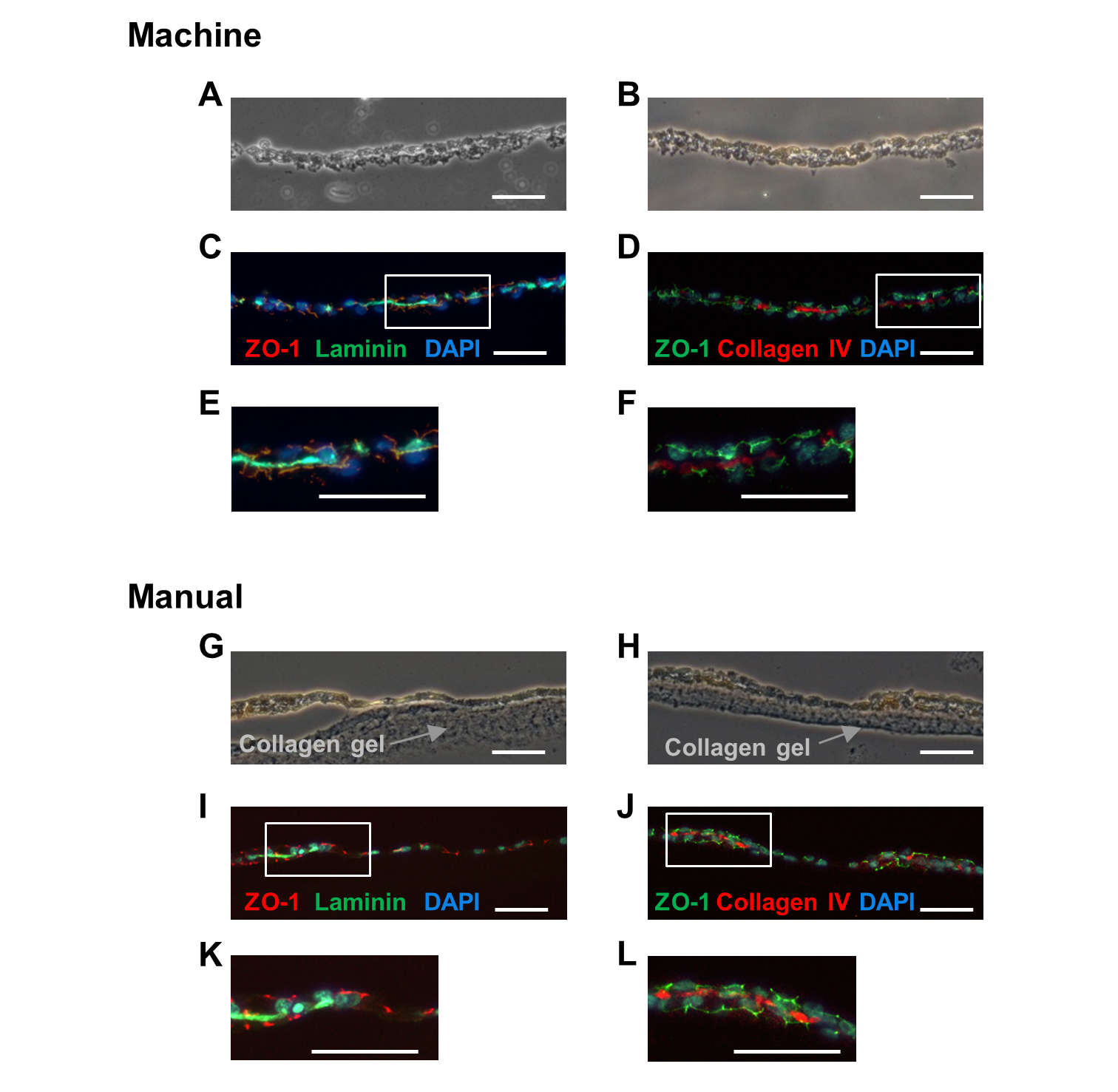

Supplement: S2 Fig — (A–F) Phase-contrast (A, B) and fluorescence (C–F) images of machine-cultured hRPE cell sheets cultured with ACE3. (A, C, E) Immunostaining for tight junction (ZO-1, red) and basement membrane (laminin, green) proteins. (B, D, F) Immunostaining for tight junction (ZO-1, green) and basement membrane (type IV collagen, red) proteins. (G–L) Phase-contrast (G, H) and fluorescence (I–L) images of manually cultured hRPE cell sheets. (G, I, K) Immunostaining for tight junction (ZO-1, red) and basement membrane (laminin, green) proteins. (H, J, L) Immunostaining for tight junction (ZO-1, green) and basement membrane (type IV collagen, red) proteins. Scale bars: 50 μm. (TIF) [file pone.0212369.s002.tif]

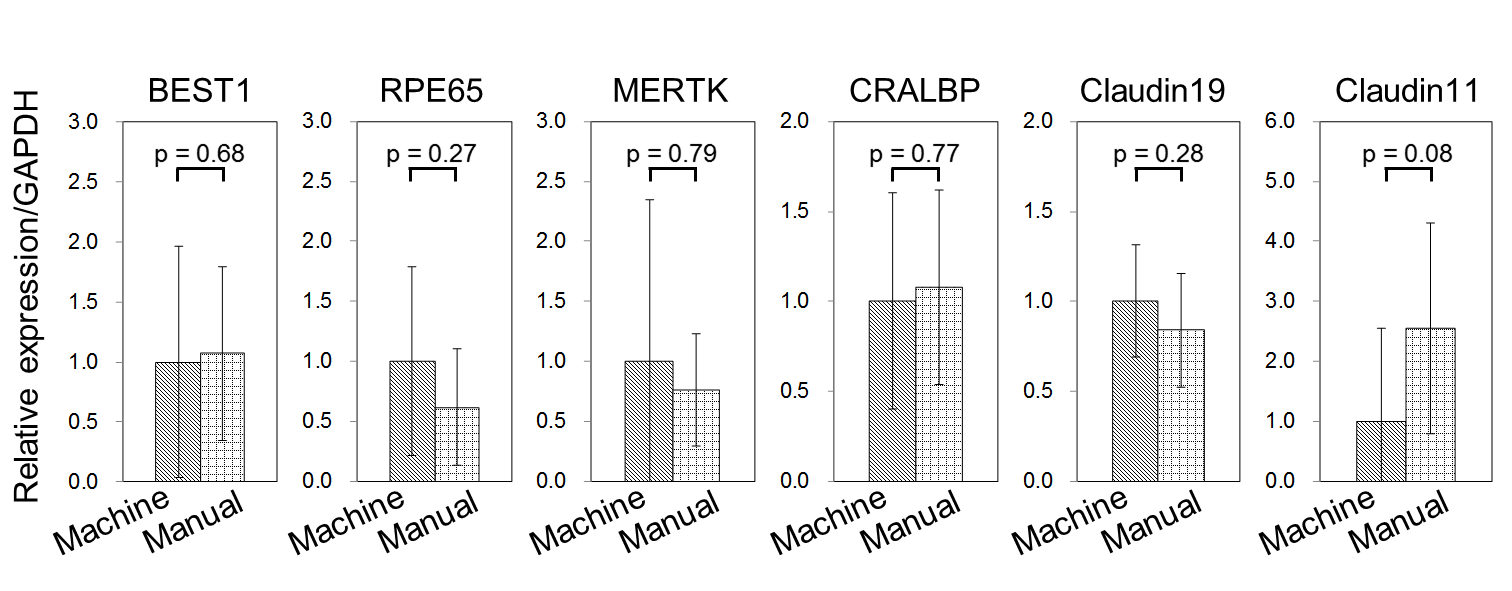

Supplement: S3 Fig — Machine cell culture, n = 5, manual cell culture, n = 4. All data are represented as the means ± SD. (TIF) [file pone.0212369.s003.tif]

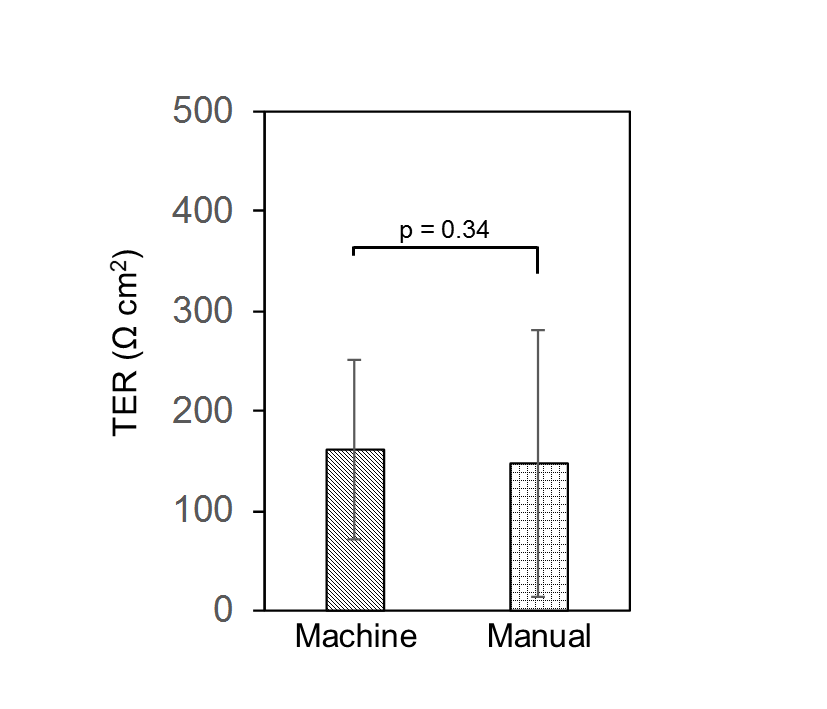

Supplement: S4 Fig — The TER values of the hRPE cell sheets were calculated by subtracting the value from inserts covered with collagen gels as a blank from those of the experimental inserts. Machine cell culture, n = 12, manual cell culture, n = 11. All data are represented as the means ± SD. (TIF) [file pone.0212369.s004.tif]

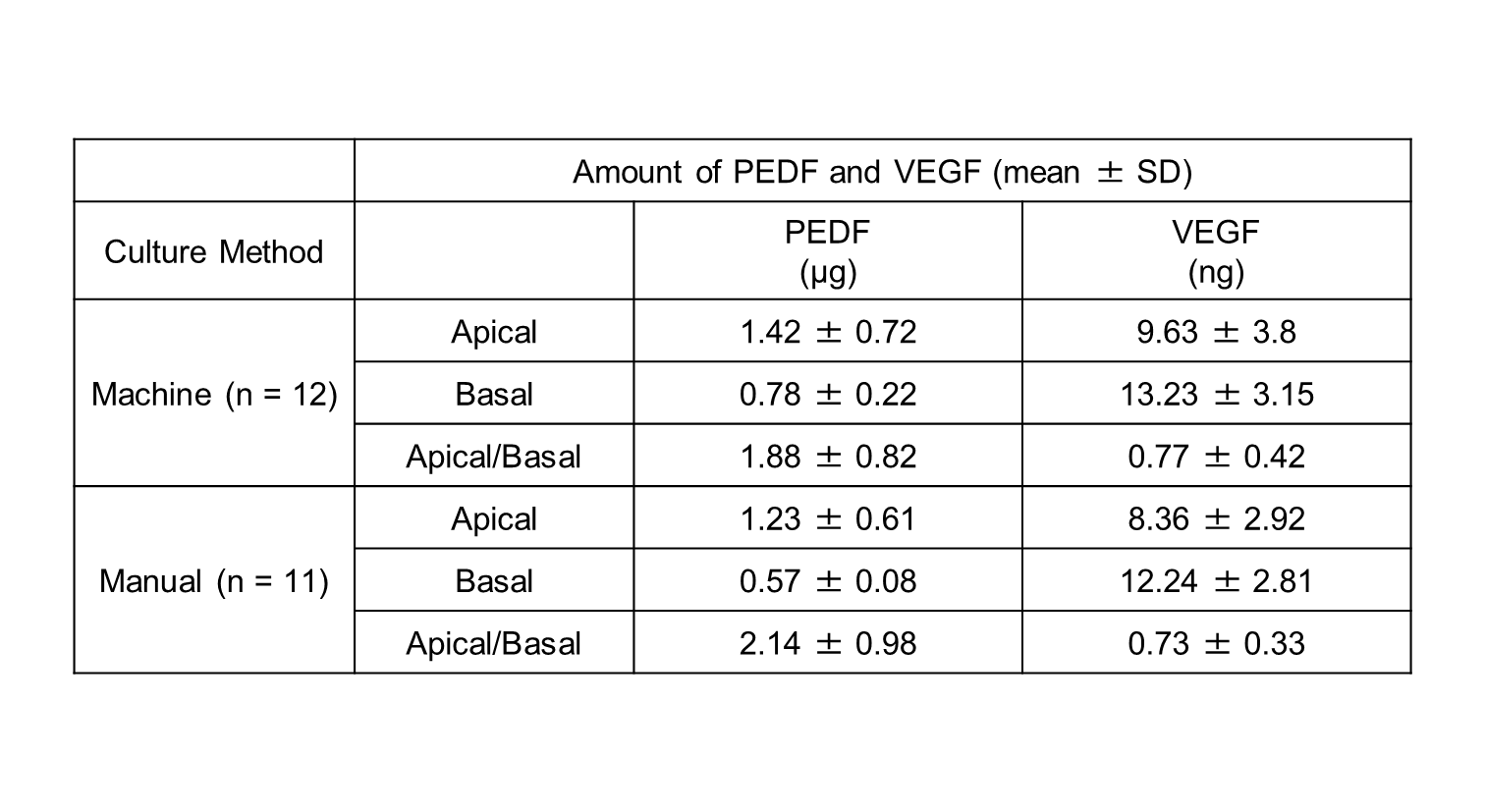

Supplement: S1 Table — (TIF) [file pone.0212369.s005.tif]
